# The Hippocratic Risk: Epidemiology of Suicide in a Sample of Medical Undergraduates

**DOI:** 10.1007/s11126-020-09844-0

**Published:** 2020-09-07

**Authors:** Livio Tarchi, Matteo Moretti, Antonio Marco Maria Osculati, Pierluigi Politi, Stefano Damiani

**Affiliations:** 1 Department of Brain and Behavioral Sciences, University of Pavia, Via Bassi 21, 27100 Pavia, PV Italy; 2 Department of Public Health, Experimental and Forensic Medicine, University of Pavia, via Forlanini 12, 27100 Pavia, Italy; 3 Unit of Legal Medicine, IRCCS Fondazione C. Mondino, via Mondino 2, 27100 Pavia, Italy

**Keywords:** Suicide, University students, Epidemiology, Mental health

## Abstract

In spite of the growing awareness on medical students’ mental health in recent years, epidemiologic literature about the topic is scarce, particularly for what concerns suicide. Here, we collected cases of suicides among medical students enrolled at a local University in Italy and compared it to the general population of the same age group. Our sample was collected using records of suicides in the city of Pavia, Italy between the years of 2014 and 2019. This record was cross-referenced with enrolment numbers to the Medical Faculty of the city in order to select cases that involved medical students A time-window of 6 years was chosen for the evaluation. The odds ratio of suicide in medical students in comparison to the general population was of 14.58 (*p* value at the exact Poisson test <0.001). We then differentiated between native Italians and students from abroad. In this case, an odds ratio of 6.81 was observed (*p* value at the exact Poisson test 0.011). Our estimated suicide rates exceeded what was previously reported for the general population. We call for a closer attention on mental health in medical students, and an increased effort in collecting objective data on the topic.

**Electronic supplementary material:**

The online version of this article (10.1007/s11126-020-09844-0) contains supplementary material, which is available to authorized users.

## Introduction

As stated by Dr. Gishen on suicide among medical students, it is central to discuss the subject more broadly and with the highest possible accuracy in order to properly investigate the risk factors and evaluate which are the most appropriate interventions to adopt [1]. A prevalent issue concerning suicide in the medical student population is the lack of objective data. Universities are often unwilling to share the numbers of involved students for both privacy reasons and legal concerns [2]. In addition, suicide-related literature often considers University students as a single population, instead of describing cases differentiating them by course [3–6]. This may be a major bias in estimating the effective suicide rates, as different degree programs appeal to distinct personality types [7, 8] and are subject to different levels of distress. The purpose of the present study is to promote scrutiny over this delicate argument by estimating the suicide rate in a sample of medical students in the city of Pavia, Italy.

## Methods

### Methods

Our sample was collected using records of suicides in the city of Pavia, Italy between the years of 2014 and 2019. Suicides were assigned to the respective student status by university records, confirmed and verifiable by the independent coverage of publicly available sources. The total number of medical students in the cohort was extrapolated from previous notices issued at the national level, as the number of medical students accepted to each Italian University in a given year is set nationwide through a governmental decree. An inquiry to the Medical Faculty was also made to infer the total number of non-Italian Medical students in the cohort, and its composition by nationality. The University of Pavia, at the moment, has two different medical programs. One is taught in English, the other in Italian. We were able to obtain the country of origin only for students enrolled to the English course. All students enrolled to the Italian course were counted as native Italians. We selected a time-window of 6 years for the evaluation, as per the availability of death registers. The sample of native Italian medical students was composed of 1427 individuals, while the sample of medical students from abroad consisted in 317 individuals.

### Procedures

Estimates of suicide rates were measured with the following formula:

$$ \frac{Completed\ Suicide\ Attempts: Sample}{length\ of\ observation}=\frac{x:100.000}{year} $$

Estimates for the general population in the same age group (15-24yo) were obtained from the WHO Mortality Database [9]. See Supplementary Fig. S1 for the country estimates of suicide rate, divided by age group and gender. Last available data for Italy was from 2015. Odds ratios were measured by the ratio of odds in suicide between samples and the general population. Considering the small sample size, rates and corresponding confidence intervals were estimated and compared (General Population in contrast with Medical students firstly, then native Italian Medical students in comparison to students from abroad). Exact Poisson test was performed when comparing i) the whole sample of medical students to the general population; ii) native Italian students with students from abroad. Fisher’s exact test was used as a control analysis, to confirm the results of the Poisson’s test.

## Results

### Number of Suicides in the Sample

In the sample of native Italian medical students, 2 suicides were reported in the population over six years. One occurred by plastic bag suffocation, in a student enrolled to the first years of the course. The second occurred by hanging and the student was enrolled to the last year of the course. Both were males. The sample of medical students from abroad, instead, reported 3 suicides over six years. Two of them occurred in students enrolled to the first years of the course, one by hanging and one by plastic bag suffocation. One of them was male, the other female. The third of them occurred in a male student enrolled to the last years of the course, by hanging. The overall sample of medical students was composed of 1744 individuals and 5 suicides occurred in this population over six years. Suicides were homogenously distributed across the considered time window (1/year except for 2014, where no suicides were recorded).

### Estimated Suicide Rates

When we considered the whole sample of medical students, we observed higher suicide rates than what previously reported for the general population in the same age group. Estimated suicide rates for our sample can be found in Table 1. A higher rate of suicides in comparison to the general population was also observed when only considering native Italian medical students, or only medical students from abroad.

**Table 1:** Estimated rates of suicides from 2014 to 2019, odds ratios and associated *p* values

	General population	Medical students	Native Italian medical students	Medical students from abroad
*N of sample*	100,000	1744	1427	317
*N of suicides over six years*	19.71*	5	2	3
*Crude rate (cases /100.000 /year)*	3.2852	47.78	23.36	105.15
*Odds ratio (Confidence Interval)*	14.58 (13.60–38.96)		6.81 (5.01–40.91)	
*p value (exact Poisson test)*	<0.001		0.011	
*p value (exact Fisher’s test)*	<0.001		0.045	

*derived

In order to estimate survival functions and the impact of different classes on the results, a Kaplan-Meier’s curve was plotted considering the duration of observations. 99.98% of the general population was retained at the end of the observation period. In contrast, 99.71% of medical students were retained; while native Italian students and students from abroad reported 99.85% and 99.05% retainment rates respectively.

As per the numerical estimates described, it is possible to appreciate differential trajectories followed by the evaluated populations.

### Odds Ratios

Odds ratios were then measured in light of the estimated suicide rates. The overall sample of medical students showed an odds ratio of 14.58 in comparison to the general population. When medical students from abroad were compared to native Italian medical students, an odds ratio of 6.81 was observed. The results were graphically reported in a histogram plot (See Table 1). Statistical significance was reached in both cases.

## Discussion

In conclusion, we observed a higher incidence of suicide in medical students in comparison to the general population. Medical students from abroad reported a considerably higher incidence than native Italian medical students, representing the group with the highest risk of suicide. This finding could be analyzed in light of results based on previous literature. First, students coming from abroad often suffer the absence of a strong social network, which is likely a protective factor against suicide [10–13]. Second, the specificity of suicide in medical students seems to rely on the same vulnerabilities already known for medical residents, such as their relatively low status in health care settings or the high stress to which they are exposed [14–17]. Third, traumas related to the medical profession could also strongly affect medical students [18, 19]. Fourth, investigations on behavioral profiles showed that medical undergraduates are more reluctant in seeking help, parallelly experiencing a high frequency of adverse life events [20].

### Limitations

Students of the medical course held in Italian were all included in the Italian nationality sample due to the lack of access to more precise data. Although a few cases of international students are enrolled to this course, the limited numerosity of this exception was deemed appropriate for exclusion. Conversely, we were able to identify the nationality for each student in the course held in English. However, inclusion within the students from abroad category was only based on these basic documents, irrespective of personal histories and narratives (for instance, second generation Italians may be strongly tied to the local culture in spite of their different nationality). A second limitation is given by the low absolute number of suicides (five in total), that may hamper the statistical reliability of the results. Larger cohorts are hence needed to confirm these results. The factors leading to suicide are multiple, dynamic and complex [21]. Many of the associated variables were not captures in this study. Finally, we were not able to record or quantify the number of suicide attempts that occurred among medical students during the same period of time, which may have allowed insightful comparisons with similar populations.

## Conclusions

In conclusion, previous literature and the present study seem to indicate a higher risk for suicide in medical students. The academic and healthcare communities are hence called to create and promote a protective environment for our future colleagues. On one hand, the millenary principle of “primum non nocere” comes back to mind, and excessive pressures should be avoided. On the other, further care in planning the course of study and associated services may allow the medical students to share and relieve their burden.

## Electronic supplementary material

ESM 1 (PDF 163 kb)

## Data Availability

The data that support the findings of this study are available from the corresponding author, S.D., upon reasonable request.

## References

[1] Gishen F (2019). Suicide among medical students. BMJ.

[2] Laitman BM, Muller D (2019). Medical student deaths by suicide: the importance of transparency. Acad Med.

[3] Mackenzie S, Wiegel JR, Mundt M, Brown D, Saewyc E, Heiligenstein E, Harahan B, Fleming M (2011). Depression and suicide ideation among students accessing campus health care. Am J Orthopsychiatry.

[4] Hawton K, Haigh R, Simkin S, Fagg J (1995). Attempted suicide in Oxford University students, 1976–1990. Psychol Med.

[5] Hamilton TK, Schweitzer RD (2000). The cost of being perfect: perfectionism and suicide ideation in university students. Aust N Z J Psychiatry.

[6] Mortier P, Auerbach RP, Alonso J, Axinn WG, Cuijpers P, Ebert DD, Green JG, Hwang I, Kessler RC, Liu H, Nock MK, Pinder-Amaker S, Sampson NA, Zaslavsky AM, Abdulmalik J, Aguilar-Gaxiola S, al-Hamzawi A, Benjet C, Demyttenaere K, Florescu S, de Girolamo G, Gureje O, Haro JM, Hu C, Huang Y, de Jonge P, Karam EG, Kiejna A, Kovess-Masfety V, Lee S, Mcgrath JJ, O’neill S, Nakov V, Pennell BE, Piazza M, Posada-Villa J, Rapsey C, Viana MC, Xavier M, Bruffaerts R (2018). Suicidal thoughts and behaviors among college students and same-aged peers: results from the World Health Organization world mental health surveys. Soc Psychiatry Psychiatr Epidemiol.

[7] Pike GR (2006). Students’ personality types, intended majors, and college expectations: further evidence concerning psychological and sociological interpretations of Holland’s theory. Res High Educ.

[8] Abedi G, Mohammadi A, Mohammadi F (2012). University students’ personality profile based on Casta and MaCrea five factor theory. International Journal of Collaborative Research on Internal Medicine & Public Health.

[9] Suicide, crude death rate per 100 000, both sexes, Italy, 2015. In: WHO Mortality Database. https://apps.who.int/healthinfo/statistics/mortality/whodpms/. Accessed 14 Aug 2020.

[10] Becker SP, Foster JA, Luebbe AM (2020). A test of the interpersonal theory of suicide in college students. J Affect Disord.

[11] Donald M, Dower J, Correa-Velez I, Jones M (2006). Risk and protective factors for medically serious suicide attempts: a comparison of hospital-based with population-based samples of young adults. Aust N Z J Psychiatry.

[12] Gould MS, Fisher P, Parides M (1996). Psychosocial risk factors of child and adolescent completed suicide. Arch Gen Psychiatry.

[13] Magne-Ingvar U, Öjehagen A, Träskman-Bendz L (1992). The social network of people who attempt suicide. Acta Psychiatr Scand.

[14] Rubin R (2014). Recent suicides highlight need to address depression in medical students and residents. JAMA.

[15] van der Heijden F, Dillingh G, Bakker A, Prins J (2008). Suicidal thoughts among medical residents with burnout. Arch Suicide Res.

[16] Jiménez-López JL, Arenas-Osuna J, Ángeles-Garay U (2015). Depression, anxiety and suicide risk symptoms among medical residents over an academic year. Rev Med Inst Mex Seguro Soc.

[17] Goebert D, Thompson D, Takeshita J, Beach C, Bryson P, Ephgrave K, Kent A, Kunkel M, Schechter J, Tate J (2009). Depressive symptoms in medical students and residents: a multischool study. Acad Med.

[18] Murray E, Krahé C, Goodsman D (2018). Are medical students in prehospital care at risk of moral injury?. Emerg Med J.

[19] Uchida C, Uchida M (2017). Characteristics and risk factors for suicide and deaths among college students: a 23-year serial prevalence study of data from 8.2 million Japanese college students. J Clin Psychiatry.

[20] Wilkinson APTJ, Gill DJ, Fitzjohn J (2006). The impact on students of adverse experiences during medical school. Med Teach.

[21] Mou D, Kleiman EM, Nock MK Proposed directions for suicide research: incorporating successful approaches from other disciplines. The British Journal of psychiatry (2020) 1–2. 10.1192/bjp.2020.58. 10.1192/bjp.2020.58 32228741

