# Supplementary material for: The Hippocratic Risk: Epidemiology of Suicide in a Sample of Medical Undergraduates
Source: Psychiatr Q. 2020 Sep 7;92(2):715–20. doi: 10.1007/s11126-020-09844-0 (PMC8110500; doi:10.1007/s11126-020-09844-0)
Supplement: Supplementary file 1 — (PDF 163 kb) [file 11126_2020_9844_MOESM1_ESM.pdf]

Supplementary Materials

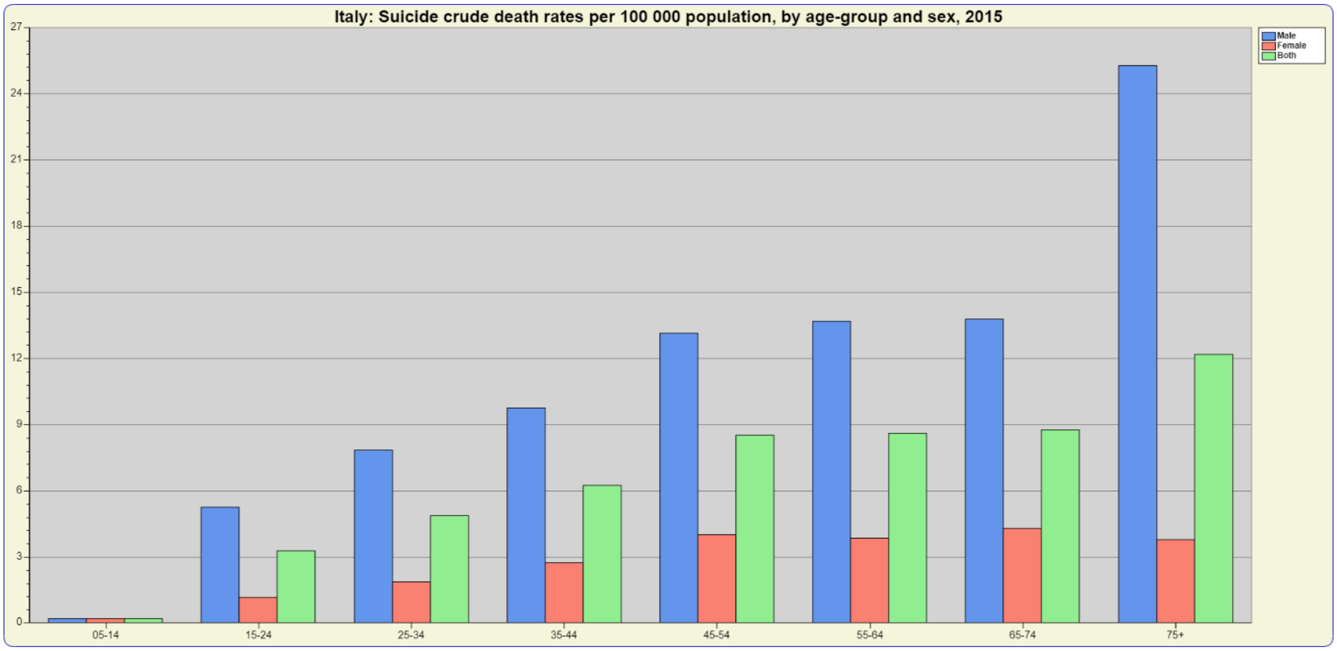

Figure S1 – Suicide death rates per 100.000, by age group and sex, Italy. Latest available from WHO Mortality Database (2015)
